# The nutritional composition and cell size of microbial biomass for food applications are defined by the growth conditions

**DOI:** 10.1186/s12934-023-02265-1

**Published:** 2023-12-11

**Authors:** Myrsini Sakarika, Frederiek-Maarten Kerckhof, Lotte Van Peteghem, Alexandra Pereira, Tim Van Den Bossche, Robbin Bouwmeester, Ralf Gabriels, Delphi Van Haver, Barbara Ulčar, Lennart Martens, Francis Impens, Nico Boon, Ramon Ganigué, Korneel Rabaey

**Affiliations:** 1 Center for Microbial Ecology and Technology (CMET), Faculty of Bioscience Engineering, Ghent University, Coupure Links 653, Ghent, B-9000 Belgium; 2 Center for Advanced Process Technology for Urban Resource recovery (CAPTURE), Frieda Saeysstraat 1, Ghent, 9052 Belgium; 3 Kytos BV, IIC UGent, Frieda Saeysstraat 1/B, Ghent, 9052 Belgium; 4 VIB-UGent Center for Medical Biotechnology, VIB, Ghent, Belgium; 5 Department of Biomolecular Medicine, Ghent University, Ghent, Belgium; 6 Proteomics Core, VIB, Ghent, Belgium

**Keywords:** Amino acid profile, Growth rate, Nucleic acid, Nutritional quality, Protein profile

## Abstract

**Background:**

It is increasingly recognized that conventional food production systems are not able to meet the globally increasing protein needs, resulting in overexploitation and depletion of resources, and environmental degradation. In this context, microbial biomass has emerged as a promising sustainable protein alternative. Nevertheless, often no consideration is given on the fact that the cultivation conditions affect the composition of microbial cells, and hence their quality and nutritional value. Apart from the properties and nutritional quality of the produced microbial food (ingredient), this can also impact its sustainability. To qualitatively assess these aspects, here, we investigated the link between substrate availability, growth rate, cell composition and size of *Cupriavidus necator* and *Komagataella phaffii*.

**Results:**

Biomass with decreased nucleic acid and increased protein content was produced at low growth rates. Conversely, high rates resulted in larger cells, which could enable more efficient biomass harvesting. The proteome allocation varied across the different growth rates, with more ribosomal proteins at higher rates, which could potentially affect the techno-functional properties of the biomass. Considering the distinct amino acid profiles established for the different cellular components, variations in their abundance impacts the product quality leading to higher cysteine and phenylalanine content at low growth rates. Therefore, we hint that costly external amino acid supplementations that are often required to meet the nutritional needs could be avoided by carefully applying conditions that enable targeted growth rates.

**Conclusion:**

In summary, we demonstrate tradeoffs between nutritional quality and production rate, and we discuss the microbial biomass properties that vary according to the growth conditions.

**Supplementary Information:**

The online version contains supplementary material available at 10.1186/s12934-023-02265-1.

## Background

The global demand for protein is expected to increase up to 78% by 2050 compared to 2017, driven by the growing population and the shift towards higher protein diets [1]. Meeting this pressing need is a major challenge due to the ever-increasing protein demand rate [2]. This threatens the conventional food production systems which, apart from not being able to meet the increasing needs, also result in overexploitation and/or depletion of resources, and environmental degradation [3]. Microbial biomass from bacteria, yeasts, filamentous fungi, or microalgae – commonly referred to as microbial protein – is an alternative protein source that has long been proposed as a solution to food scarcity [4] and that can be more sustainable than conventional food protein sources [5]. Microbial biomass is also nutritionally valuable, with characteristics that meet the nutritional requirements of humans [6].

The composition of a food source affects its nutritional value. Additionally, the macromolecular composition of microorganisms changes in function of their environment, and, at non-inhibiting cultivation conditions (e.g. optimal pH and temperature range), is related to the availability of carbon, energy, macro- and micro-nutrients [7], which will be referred to as substrate throughout this work. As the entire microbial biomass, not just its protein, is considered for consumption [6], these environmental and cellular composition relationships have a major impact on the final product quality and production efficiency. More specifically, substrate availability, which is linked to the growth rate at non-inhibiting conditions, can affect the cellular content of protein, nucleic acids (NA), carbohydrates, lipids and polyhydroxyalkanoates (PHA) (Cooney et al., 1976; Du et al., 2000). Considering that bacterial biomass can contain up to 25% NA [10] and the lack of uricase enzyme in humans, a NA-rich diet can cause kidney stone formation [11], therefore, reducing the NA content of microbial biomass prior to its use as food (or feed) is crucial. High NA content could potentially require more intensive downstream processing, which already represents 13–45% of the total electricity for dry microbial biomass production [12] and can result in 20–30% product losses [10] thereby potentially compromising the sustainability of microbial biomass as a food source. At the same time, the availability or limitation of a specific substrate (e.g. carbon, nitrogen or other micro/macro-nutrient) can cause changes in a range of metabolic functions [13], which, can lead to a broad range of alterations in the macromolecular composition of microbial cells, depending on the limiting substrate. Furthermore, the impact of changes in the macromolecular composition on the protein profile and amino acid (AA) distribution remains unknown and could significantly affect the nutritional quality and properties of microbial biomass. The cultivation conditions also affect the size of microbial cells [14], which in turn affects the harvesting, as smaller cells result in a lower microbial biomass recovery, impacting the yield of the process [15]. Thus, careful selection of operational conditions for microbial biomass production is crucial as it can significantly affect its nutritional quality, environmental footprint, and economics.

Processes targeting microbial biomass production will likely be performed under a (semi-)continuous mode [16], with the substrate supplied based on the demand to prevent substrate inhibition or limitation, to favor the economics and minimize their environmental impact. While the concentration of substrate(s) remains relatively stable during continuous cultivation, it varies during batch and semi-continuous processes (e.g. fed-batch, depending on the operational choices) [17], thereby variably affecting the macromolecular composition of the produced biomass. It should also be noted that repeated fed-batch has been proposed as the most promising cultivation mode for microbial biomass production [16]. Current research in microbial biomass production mostly focuses on assessing the quality of the biomass only at the end of a batch growth and often does not consider how limiting substrate conditions shape cell composition and product quality. At the same time, studies frequently prioritize maximizing the protein content or productivity, neglecting other nutritionally relevant parameters such as the presence and concentration of specific proteins, AA profile or NA content.

**Fig. 1:**
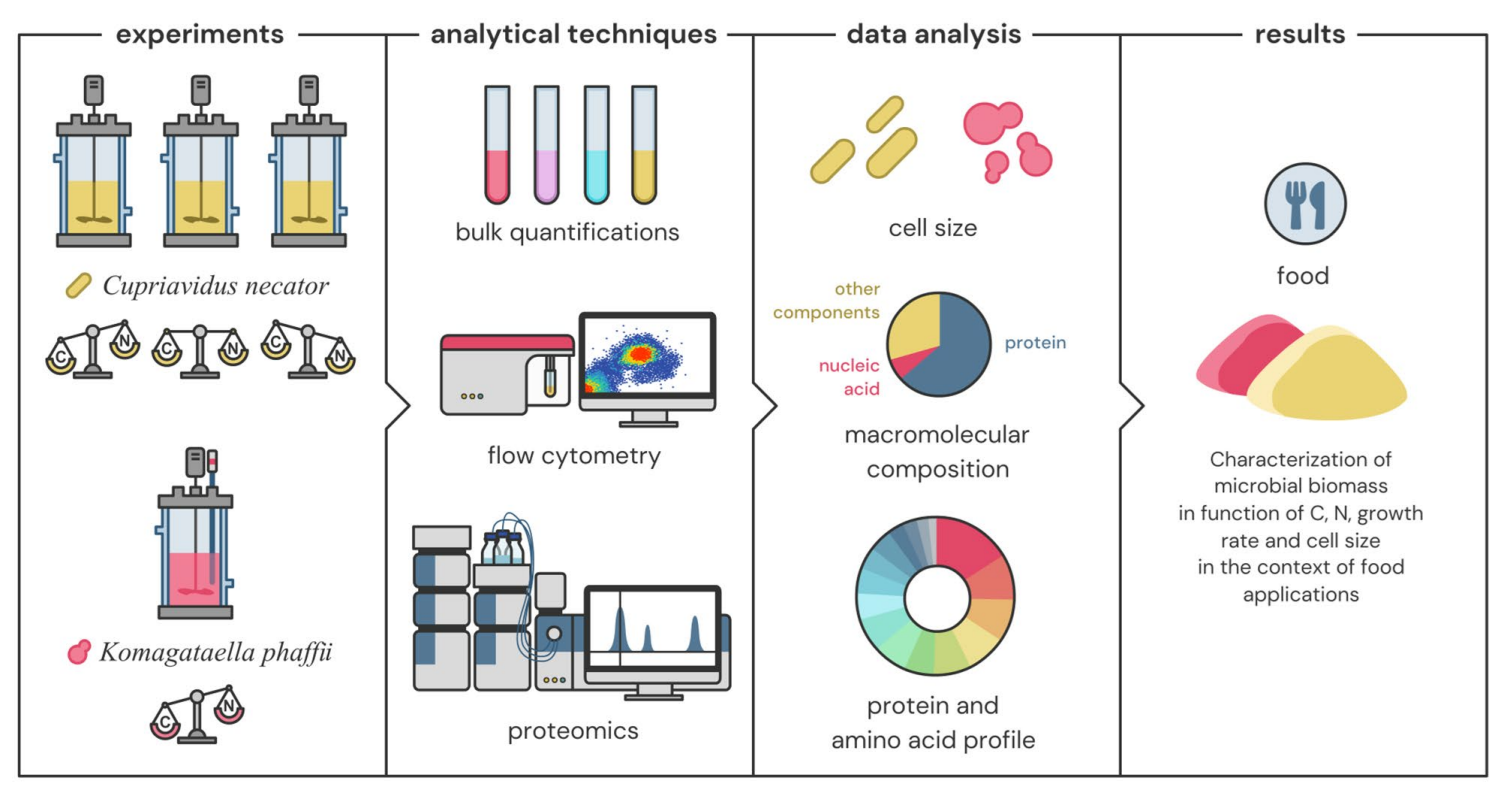
Methodology followed for evaluating the characteristics of microbial biomass for food applications based on substrate availability, growth rate, and microbial cell size

The aim of this work was to address these fundamental knowledge gaps by identifying the links between the macromolecular composition, protein and AA profile of growing microbial cells and (i) the availability of substrate (carbon and nitrogen), (ii) the growth rate and (iii) the cell size, using a combination of well-established bulk analyses and high-resolution analytical techniques such as flow cytometry and mass spectrometry (MS)-based proteomics (Fig. 1). The results obtained using the biotechnologically relevant *Cupriavidus necator* and *Komagataella phaffii* were analyzed in the context of microbial biomass production for food applications. The key tradeoffs between production rate, substrate availability, nutritional microbial biomass quality, and microbial cell size were identified. Changes in the proteome allocation were studied, and the AA profile of the whole biomass and the different cellular components was assessed. The cultivation conditions that enable higher product quality for food applications were deduced based on these results, as these parameters could impact the properties of microbial biomass and the processing required to convert it into edible food thereby substantially affecting the sustainability and economics of microbial biomass production.

## Materials and methods

### Cultures and pre-cultivation media

*C. necator* (previously known as *Ralstonia eutropha*) LMG 1199 was purchased from the Belgian coordinated collection of microorganisms (BCCM/LMG, Ghent, Belgium). *K. phaffii* ATCC 76273 was obtained from the American type culture collection (ATCC, Virginia, United States). The pre-cultures of *C. necator* and *K. phaffii*, were grown on nutrient broth (Carl Roth) and Sabouraud broth (ATCC medium 28), respectively, using a temperature-controlled (28 °C) orbital shaker (120 rpm). Fully grown cultures (harvested during the late exponential growth phase) were centrifuged at 6,603 g for 5 min, washed with phosphate buffer saline (PBS) and were then used for the subsequent experiments.

### General methodology

This study aimed to examine the link between growth rate and substrate concentration with the macromolecular composition of two model organisms that have obtained the qualified presumption of safety (QPS) status, *C. necator* and *K. phaffii*. The cultures were grown in batch, initially under substrate abundance and next under carbon (*C. necator* and *K. phaffii*), nitrogen as well as concurrent carbon and nitrogen limitation (*C. necator*) (Fig. 1), where substrate limitation is defined as the concentration of a substrate (here carbon or nitrogen) that restricts the microbial growth rate [18]. Batch growth was selected since it is governed by the same principles of unsteady state operation as the commonly used for biomass production fed-batch operation, where the growth rate is the result of substrate availability [19].

Sampling was conducted regularly to obtain a comprehensive understanding of the macromolecular composition of the cultures, including proteins, carbohydrates, NA, PHA, and neutral lipids, and their link with growth rate and substrate concentration. Proteomics analysis was used to determine protein and AA profiles. We followed up the observed growth rate and the content of organic compounds (expressed as chemical oxygen demand (COD), similar to electron equivalents (eeq), with 1 eeq = 8 gCOD) and nitrogen in the form of ammonium (NH_4_^+^). For simplicity, COD and ammonium will be referred to as carbon and nitrogen, or substrates throughout the manuscript.

### Bioreactor experiments

*C. necator* was grown in batch in modified ammonium mineral salts (AMS) medium containing vitamins and 2 g/L fructose. To achieve each limitation at the deceleration phase of the batch experiment – carbon, nitrogen and concurrent carbon and nitrogen – the nitrogen concentration was adapted. The culture was inoculated at 10% (v/v) at an initial optical density (OD) of 0.16 ± 0.02, in 1 L glass bioreactors with 0.8 L working volume. The bioreactors were equipped with a heating jacket to maintain the temperature at 28 °C, the initial pH was 6.8, and a mixing intensity of 500 rpm was achieved using a magnetic stirrer. The aeration rate was set at ca. 1 vvm using filter-sterilized air (0.2 μm pore size, Millipore, Merck) and samples (ca. 12 mL) were taken every two hours. The experiments were terminated after two consecutive similar OD values, anticipating this would indicate that the stationary phase was reached. Experiments were performed in quadruplicate, and mean values and standard deviation are presented.

Batch experiments using *K. phaffii* were performed in a 2 L glass bioreactor with 1 L working volume and a heating jacket to maintain the temperature at 30 °C. Carbon limitation was targeted at the deceleration phase of the experiment. The pH was set at 7.0 and automatically corrected using autoclaved NaOH 5 M. A mixing intensity of 500 rpm was achieved using an impeller, while the aeration rate was set at 2 vvm using filter-sterilized air (0.2 μm pore size, Millipore, Merck). The mineral medium used here was adapted from Delaney et al. [20], due to better performance compared to other media tested. Methanol was used as a substrate, at an initial concentration of 5 g/L. The cultures were inoculated at 10% (v/v) at an initial OD of 0.24 ± 0.002. Samples (ca. 12 mL) were taken three times per day for 2 days. This experiment was performed in duplicate and mean values are presented.

Samples for protein and proteomics analyses (2 mL each) were centrifuged at 20,817 g for 5 min at room temperature, washed once with PBS, and the biomass pellet was stored at -20 °C until analysis. The supernatant of the first centrifugation was filtered (0.2 μm PVDF filters, Chromafil®) and stored (-20 °C) separately for later determination of fructose, methanol, ammonium and metabolite (organic acids and/or alcohols) concentrations. Samples for total suspended solids (TSS) analysis (ca. 5 mL) were stored at -20 °C as such. Sample treatment for flow cytometry is described in below.

### Bulk analytical techniques

The growth of *C. necator* and *K. phaffii* was monitored by measuring the OD at 600 nm using a spectrophotometer (Spectronic 22, Thermo Scientific). The cell dry weight (CDW) was quantified as TSS using hydrophilic nylon membrane filters of 0.2 μm pore size (GNWP04700; Millipore; Merck) for *C. necator* and glass fiber filters of 0.7 μm pore size (AP4004705; Millipore; Merck) for *K. phaffii*, in suspensions of 4–15 mL depending on the biomass density. The pH was measured for each time point using a pH sensor (ORION911600, Thermo Scientific). Ammonium was determined using an ion chromatograph (930 Compact IC Flex; Metrohm, CH), equipped with a Metrosep A Supp 5- 150/4.0 column, a Metrosep A Supp 4/5 guard column/4.0 and an 850 IC conductivity detector (Metrohm, CH). The cations were eluted at a flow rate of 0.7 mL/min using 1.7 mM HNO_3_ (2 M; ThermoFisher Scientific) and 1.7 mM 2,6-pyridinedicarboxylic acid (≥ 99.5%; Sigma-Aldrich). Total cellular protein was analyzed in triplicate using Pierce™ bicinchoninic acid (BCA) protein assay kit (ThermoFisher Scientific) with bovine serum albumin as a standard. Total cellular carbohydrates were determined colorimetrically using the Josefsson [21] method with D-glucose (≥ 99.5%, Carl Roth) as a standard. Organic acids, alcohols and residual fructose were determined using an HPLC (Prominence-i LC 2030 Plus, Shimadzu) equipped with a refractive index detector (RID-20 A, Shimadzu) and an Aminex HPX-87 H column with 9 μm particle size (7.8 × 300 mm, Bio-Rad), at a column temperature of 41 °C. Sulfuric acid (5 mM H_2_SO_4_) was eluted at a flow rate of 0.6 mL/min. The concentration of all organic compounds in the medium was summarized and expressed as COD (g/L).

### Proteomics analysis

#### Protein extraction and digestion

Cell pellets of 2–6 mL suspensions depending on the protein content, were homogenized in 100 µl urea lysis buffer (8 M urea, 20 mM HEPES pH 8.0). Half of the lysate was diluted twice in lysis buffer containing 10% sodium dodecyl sulfate (SDS) and 100 mM triethylammonium bicarbonate (TEAB), at pH 8.5. The resulting lysates (5% SDS, 4 M urea, 10 mM HEPES, 50 mM TEAB) were transferred to a 96-well PIXUL plate and sonicated with a PIXUL Multisample sonicator (Active Motif) for 60 min with default settings (Pulse 50 cycles, PRF 1 kHz, Burst Rate 20 Hz). After centrifugation of the samples for 15 min at 2,204 × g at room temperature to remove insoluble components, the protein concentration was measured by BCA assay and 50 µg of protein was isolated from each sample to continue the protocol. Proteins were reduced by the addition of 15 mM dithiothreitol and incubation for 30 min at 55˚C and then alkylated by the addition of 30 mM iodoacetamide and incubation for 15 min at room temperature in the dark. Phosphoric acid was added to a final concentration of 1.2%, and subsequently, samples were diluted 7-fold with binding buffer containing 90% methanol in 100 mM TEAB, pH 7.55. The samples were loaded on the 96-well S-Trap™ plate (Protifi), and a Resolvex® A200 positive pressure workstation (Tecan Group Ltd) was used for semi-automatic processing. After protein binding, the S-trap™ plate was washed three times with 200 µL binding buffer. A deep well plate was placed below the S-Trap™ plate, and 125 µL 50 mM TEAB containing 1 µg trypsin (1/100, w/w) was added for digestion overnight at 37 °C. Peptides were eluted three times using the Resolvex® A200 workstation, first with 80 µL 50 mM TEAB, then with 80 µl 0.2% formic acid (FA) in water and finally with 80 µl 0.2% FA in water/acetonitrile (ACN) (50/50, v/v). Eluted peptides were dried completely by vacuum centrifugation, re-dissolved in 100 µl 0.1% trifluoroacetic acid (TFA) in water/ACN (95/5, v/v) and reapplied to the S-trap™ plate placed above a new deep well plate. Using the Resolvex® A200 workstation, peptides were pushed through the S-trap™ material and finally eluted with 80 µL 0.2% FA in water/ACN (50/50, v/v). The combined eluates were transferred to HPLC inserts and dried in a vacuum concentrator.

#### LC-MS/MS analysis

Peptides were re-dissolved in 20 µL loading solvent A (0.1% TFA in water/ACN (98:2, v/v)) and 1.5 µL was injected for LC-MS/MS analysis on an Ultimate 3000 RSLC nanoLC (Thermo Scientific, Bremen, Germany) in-line connected to a Q Exactive HF Biopharma mass spectrometer (Thermo) equipped with a Nanospray Flex™ Ion Source. Trapping was performed at 10 µL/min for 4 min in loading solvent A on a 20 mm trapping column (made in-house, 100 μm internal diameter, 5 μm beads, C18 Reprosil-HD, Dr. Maisch, Germany) and the sample was loaded on a 200 cm long micropillar array column (PharmaFluidics) with C18-endcapped functionality mounted in the column oven of Ultimate 3000 at 50 °C. For proper ionization, a sharpened fused silica ESI emitter (10 μm internal diameter, Fossiliontech) was connected to the µPAC™ outlet union and a grounded connection was provided to this union. Peptides were eluted by a non-linear increase starting from 1% MS solvent B (0.1% FA in water/ACN (2:8, v/v)) reaching 55% MS solvent B in 100 min and 70% MS solvent B in 125 min, first at a flow rate of 500 nL/min for 15 min, then at 300 nL/min, followed by a 5-min wash at 70% MS solvent B and re-equilibration with 99% MS solvent A (0.1% FA in water). The MS was operated in data-dependent mode, automatically switching between MS and MS/MS acquisition for the 16 most abundant ion peaks per MS spectrum. Full-scan MS spectra (375-1,500 m/z) were acquired at a resolution of 60,000 in the Orbitrap analyzer after accumulation to a target value of 3,000,000. The 16 most intense ions above a threshold value of 13,000 (minimum AGC of 1,000) were isolated for fragmentation at a normalized collision energy of 28%. The C-trap was filled at a target value of 100,000 for a maximum of 80 msec and the MS/MS spectra (200-2,000 m/z) were acquired at a resolution of 15,000 in the Orbitrap analyzer with a fixed first mass of 145 m/z. Only peptides with charge states ranging from + 2 to + 6 were included for fragmentation and the dynamic exclusion was set to 12 s. QCloud was used to control instrument longitudinal performance during the project.

#### Flow cytometry

All flow cytometry acquisitions were performed using Attune™ NxT (Invitrogen™, ThermoFisher Scientific) with BRxx configuration equipped with 488 nm (blue/BL) and 635 nm (red/RL) lasers and default optical filters. The acquisition was performed using 96-well plates at a final working volume of 200 µL. The flow rate was set at 100 µL/min. A fluorescence threshold was set based on the dye used (see below) and event rates were generally kept below 8,000 events/sec by diluting in filter-sterilized PBS (ThermoFischer Scientific). An initial lead-time of 15 s was not recorded to stabilize the flow and the acquisition was stopped after 75 µL of volume was recorded. The events and fluorescence stability over time were closely monitored for acquisition-level stability.

#### Total, intact, and damaged cell concentrations

Total cell concentration and intact cell concentration were determined by staining with SYBR Green I (SG) and SYBR Green I/Propidium Iodide (SGPI) (Invitrogen™, ThermoFischer Scientific), respectively. The trigger was the green fluorescence in the BL1 (530/30BP) channel. SG was detected on BL1 (530/30BP), while propidium iodide was detected on BL3 (695/40BP). The procedure contained the following steps: (i) Dilution of untreated sample to achieve event rates ranging between 200 and 6,000 events/sec; (ii) Staining at 1% (2 µL) using SG for total cell concentrations or SGPI for intact and damaged cell concentrations; (iii) Incubation at 37 °C for 20 min; (iv) Flow cytometry analysis.

#### Nucleic acid intensity determination with SYTO^™^ 62 stain

Using the Thermo SYTO™ Red fluorescent NA stain sampler kit (Invitrogen™, ThermoFischer Scientific), we assessed staining efficiency and stability for SYTO™ 60–62 and concluded that SYTO™ 62 offered the best tradeoff between low noise and stain stability. SYTO™ 62 was therefore used as a counterstain for PHA and neutral lipid as well as for NA intensity determination due to its higher affinity for RNA compared to SG. The NA intensity was used as an approximation of the NA content in the microbial cells, assuming a linear relationship.

#### Polyhydroxyalkanoate (PHA) and Neutral lipid intensity determination with BODIPY^™^ 493/503 stain

The procedure from Karmann et al. [22] was adapted to measure the intracellular PHA intensity. The resulting procedure contained the following steps: (i) A suspension of 1 mL was centrifuged at 5,000 g for 5 min; (ii) The supernatant was discarded, the biomass was washed with PBS and the sample was centrifuged again; (iii) Finally, the sample was fixated using 0.8% paraformaldehyde (PFA; Sigma-Aldrich), at 4°C for 1 h; (iv) Dilution to ca. 2.1 · 10^4^ ± 1.5 · 10^4^ cells/mL (based on total cell concentrations using SG) using PBS containing 5 mM EDTA; (v) Addition of 2 µL SYTO™ 62 (stain diluted in DMSO in final concentration of 0.5 µΜ in 200 µL diluted sample); (vi) Addition of 2 µL BODIPY™ 493/503 (Invitrogen™, ThermoFisher Scientific; diluted in DMSO at a concentration of 10 µg/mL in 200 µL diluted sample); vii. Incubation for 5 min at 30 °C; (viii) Flow cytometry analysis. During data analysis, PHA-positive cells were determined by a triple-threshold with a (low) SSC-H threshold, followed by an appropriate threshold on the SYTO™ 62 fluorescence (RL1:670/14BP) and a minor threshold on the fluorescence on the PHA-selective stain (BL1:530/30BP). The PHA and neutral lipid intensity was used as an approximation of the content of these compounds in the microbial cells, assuming a linear relationship.

### Data analysis and calculations

Flow cytometry data (in FCS format) was exported from the Attune NXT software and processed using R (v4.1.1). Data was loaded and processed using the R package flowCore (version 2.4.0) and hierarchical gating was applied using flowWorkspace (version 4.4.0). Singlets were discriminated from aggregated cells along the primary fluorescence channel for the SG NA stain to accurately determine the cell concentration. The flowAI R package (version 1.24.0) was used for event-level quality control. Based on dynamic range and fluorescence stability, aberrant events were removed from the analysis using SYPRO™ Orange and BODIPY™ 493/503 stains. It should be noted that a linear relationship between intensity and content (for NA and PHA/neutral lipids) is assumed. Cell diameters were estimated using the proprietary KytoFlow software (Kytos BV, Belgium) and median values are presented as an approximation of the cell size.

Prior to analyzing the samples of this work, samples from the cultures used here were analyzed with the gold standard method by an external accredited commercial laboratory, to evaluate if bottom-up proteomics yields the same results as a hydrolysate (Fig. S1). The LC-MS/MS data were searched using the MSFragger version 3.5 against their respective databases for *Komagataella phaffii* (UniProt ID: UP000094823) and *Cupriavidus necator* (UniProt ID: UP000006798). The standard contaminant list from MSFragger was added to each search database. Peptides with up to two missed cleavages were allowed in the search. Search parameters were set to 20 ppm precursor ion mass tolerance and 20 ppm fragment mass tolerance. Carbamidomethylation of cysteine was set as a fixed modification. Methionine oxidation and N-terminal protein acetylation were set as variable modifications. All remaining search parameters were set to default for a closed search. Peptide spectrum match (PSM) rescoring was performed with MSBooster and Percolator version 3.6. The acquired PSMs that met the 1% false discovery rate threshold were used for protein inference with ProteinProphet v. 4.2.1. The PSM count per protein was then normalized by protein length and this normalized PSM count was then used as quantification value per protein. The AA content (g AA/g protein) was then quantified using the product of protein quantification numbers with their respective AA count. The gene ontology (GO) terms were retrieved based on the protein IDs using the UniProt batch retrieve tool (https://www.uniprot.org/id-mapping) via an in-house script. Next, the proteins to which no cellular component GO identifier was assigned were filtered out and the EMBL QuickGO REST API (https://www.ebi.ac.uk/QuickGO/api/index.html) was used to retrieve all children of the GO terms “cellular anatomic entity” (GO:0110165), “protein containing complex” (GO:0032991) and “ribosome” (GO:0005840). For each cellular component GO ID, we performed an API call to retrieve its ancestors with the “part of” and “is a” relationship, which were subsequently joined with the original list of cellular component GO ID and only unique IDs were retained. This list was then mapped against the previously created reference. If no matches were found the list of unique GO IDs was returned and their term information was retrieved using the EMBL API. If more than one match was found, the ontology relations were checked, to only retain terms that had no cross-ontology relations with the others. The results were further simplified by grouping all children of the protein containing complex GO term as “protein containing complexes” and grouping all membrane-associated non-matching terms under “membrane”. The retained identified peptides and their associated measurement data are referred to as the “annotated proteome”. The (razor) protein intensities of each term were summed, and the top 3 entries are used.

Correlograms were constructed based on Spearman’s ρ at a significance level of 5% to identify general trends, using R version 4.2.2.

## Results and discussion

### The macromolecular composition of a microbial cell is linked to substrate availability

**Fig. 2:**
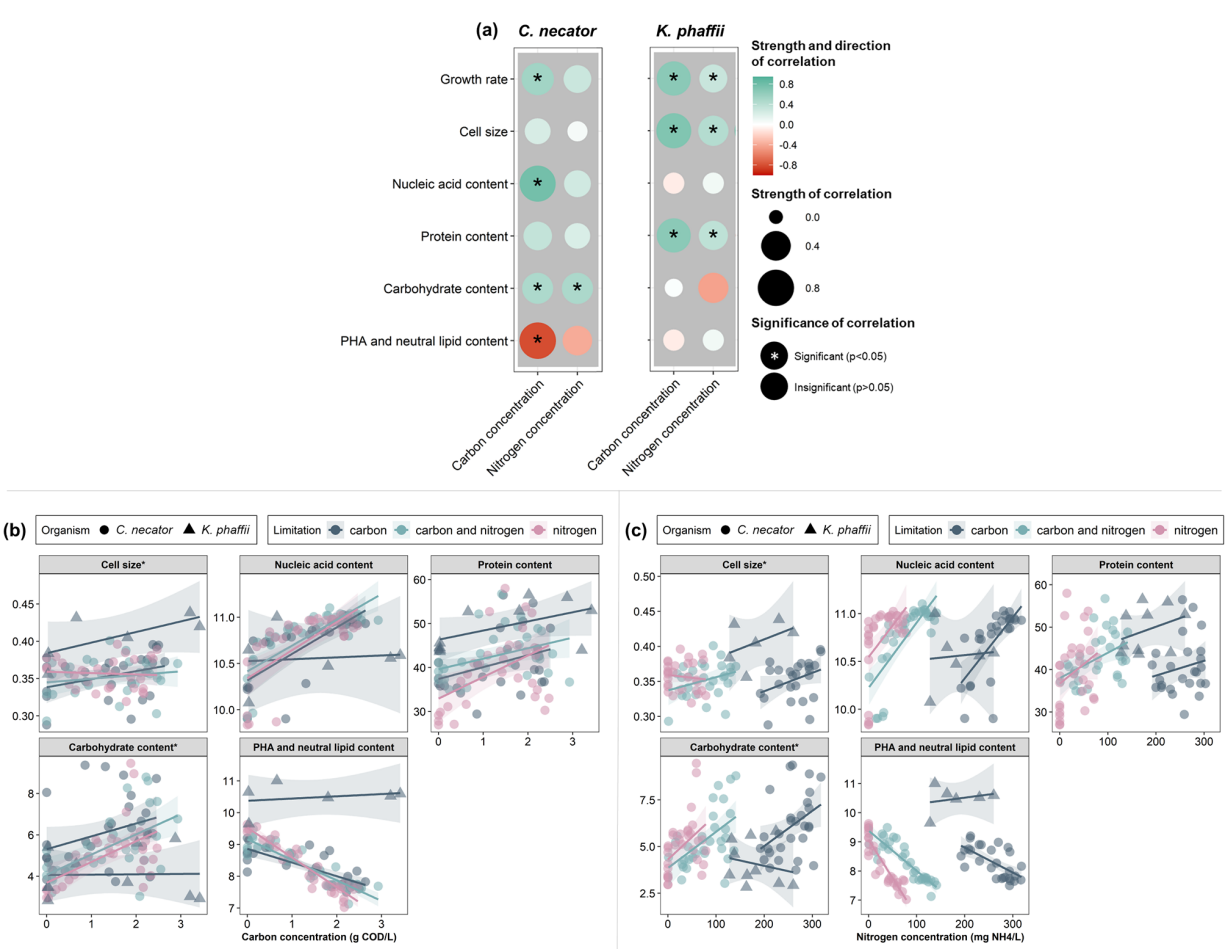
(**a**) Correlogram for the relationship between growth rate (h^− 1^), cell size (diameter, in µm) and macromolecular cell composition (nucleic acid (A.U.), protein (g protein/g CDW), carbohydrate (g carbohydrate/g CDW), PHA and lipid (A.U.) content) in function of substrate (carbon (g COD/L) and nitrogen (g NH_4_^+^/L)) concentration for *C. necator* and *K. phaffii*. Macromolecular composition and cell size in function of (**b**) growth rate and (**c**) cell size throughout the growth of *C. necator* and *K. phaffii*. The shaded area indicates the 95% confidence interval of a linear model fit. *The cell size and carbohydrate content of *K. phaffii* were divided by 5 to allow for better representation of the trends from both organisms in the same graph

The macromolecular composition and cell size were impacted by carbon and nitrogen availability, with the most significant associations noted for NA, carbohydrates, PHA and neutral lipid content for *C. necator* and with cell size and protein content for *K. phaffii* (Fig. 2). Both *C. necator* and *K. phaffii* had higher protein content and larger cells at higher substrate concentrations (Fig. 2(a)). Nitrogen limitation led to generally larger cells and higher PHA and neutral lipid content in *C. necator*, while it remained relatively unchanged for *K. phaffii* (Fig. S2). This is in line with the fact that storage compounds are largely affected by nutrient limitation. PHB (a sub-group of PHA) accumulation is conventionally stimulated *via* the limitation of nutrients such as nitrogen [9], which typically limits the cell replication. Indeed, here a negative correlation was established between PHA content and cell productivity under substrate-limiting conditions (Fig. S4(b)). The opposite relationship compared to PHA was observed for the carbohydrate content in *C. necator*, where higher substrate concentrations and higher cell productivity generated cells with higher carbohydrate content (Fig. S4(b)), while the carbohydrate content remained relatively constant for *K. phaffii*.

It could be possible, by limiting a specific substrate, to compromise the cell growth but favor the synthesis of desired intracellular macromolecules [7]. For instance, carbon limitation can lead to a limited supply of energy (in heterotrophs) and carbon for biosynthesis; limitation in nitrogen or sulfate can result in lower protein synthesis; in contrast, limited phosphorus, potassium or magnesium supply can lead to lower NA content. Here, nitrogen concentration was positively correlated with the protein content and negatively correlated with the NA content (Fig. 2). Additionally, the NA content significantly increased with increasing carbon concentrations in *C. necator* but showed a weak negative correlation in *K. phaffii*. When both carbon and nitrogen were the limiting substrates, the macromolecular composition of the cells was within the limits established by the single-substrate limitation, which is in agreement with previous findings [7, 23]. Nevertheless, under carbon limitation, the concentration of intracellular carbon and energy intermediates NADH and ATP can drop as these species are consumed faster than replaced [9]. Therefore, even though substrate limitation can serve as means to tune the biomass characteristics, it should not be prolonged so that essential metabolic functions are not restricted. Considering the variable effects based on the limitation of different substrates, their availability should be chosen based on the targeted nutritional quality of the microbial biomass.

### The macromolecular composition of a microbial cell is interlinked to its growth rate

Considering that the growth rate is significantly impacted by substrate availability (Fig. 2) and that the limitation of different substrates has a distinct effect on the nutritional quality of microbial biomass, as discussed in the previous section, the macromolecular composition at different growth rates should be assessed separately for the different limiting substrates.

**Fig. 3:**
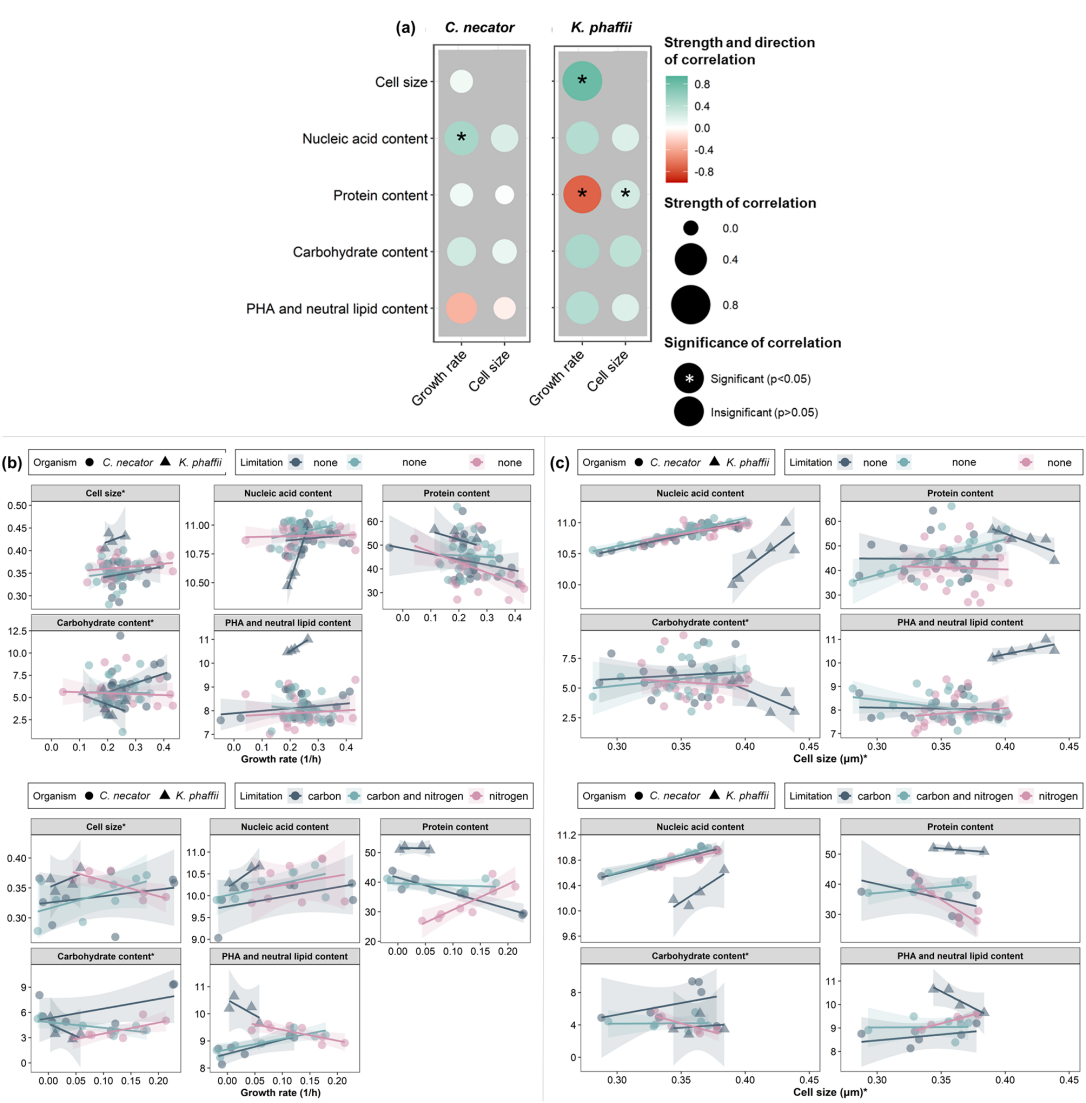
(**a**) Correlogram for the relationship between cell size (µm) and macromolecular cell composition (nucleic acid (A.U.), protein (g protein/g CDW), carbohydrate (g carbohydrate/g CDW), PHA and lipid (A.U.) content) in function of growth rate and cell size for *C. necator* and *K. phaffii*. Macromolecular composition and cell size in function of (**b**) carbon and (**c**) nitrogen concentration throughout the growth of *C. necator* and *K. phaffii* under substrate-abundant (top) and substrate-limiting conditions (bottom). The shaded area indicates the 95% confidence interval of a linear model fit. *The cell size and carbohydrate content of *K. phaffii* were divided by 5 to allow for better representation of the trends from both organisms in the same graph

The most prominent correlation between the growth rate and the macromolecular composition was its positive relationship with the NA content regardless of the limiting substrate (Fig. 3), with a plateau observed at a growth rate of *C. necator* greater than 0.3 h^− 1^ (Fig. 3(b)). This increase is associated with intensified RNA synthesis at higher growth rates [7, 8, 24], as the DNA content remains relatively stable throughout the different growth rates [8, 25]. For instance, the RNA content of *Acinetobacter calcoaceticus* doubled (from 12 to 27%) with a 9-fold increase in the growth rate, irrespective of the substrate [26].

The protein content decreased with increasing growth rate for both *C. necator* and *K. phaffii* (Fig. 3). Exception to that was the growth of *C. necator* under concurrently carbon and nitrogen-limited conditions, where an increase in protein content with rising growth rate was observed (Fig. 3(b), bottom). Under substrate-limiting conditions, the protein content of *C. necator* increased with an increase in nitrogen concentration and cell productivity (Fig. S4(b)). Upon further analysis, a negative correlation was observed between protein content and the growth rate of *C. necator* when substrate was abundant (Fig. 3(b), top). A negative correlation was also observed when substrate availability was limited, except for nitrogen-limited cells, where a positive correlation was noted (Fig. 3(b), bottom). Studies on the carbon-limited bacteria *A. calcoaceticus* [26] and *Pseudomonas* sp. [27] showed a 9–11% decrease in protein content with 7.1-9.7-fold increase in growth rate. Decreased protein content with increasing growth rate was also seen in two bacteria (*Methylorubrum extorquens*, *Corynebacterium glutamicum*) and three yeasts (*Cyberlindnera saturnus*, *Metschnikowia pulcherrima* and *Wickerhamomyces anomalus*) [23].

The growth rate also correlated with the intracellular storage compound content (e.g. carbohydrates, PHA and lipids) under substrate-limiting conditions, with low growth rates promoting their intracellular accumulation (Fig. 3(b)). However, potential changes in the content of storage compounds depend on the ability of a specific microorganism to produce them, as well as on the substrate availability. For instance, *C. necator* is a well-known PHB producer [28], while *K. phaffii* stores intracellular neutral lipids [29]. The PHA content of carbon-, or concurrent carbon and nitrogen-limited *C. necator* cells increased with an increased growth rate, while the nitrogen-limited cells presented the opposite trend (Fig. 3(b), bottom). Under substrate-limiting conditions, the correlation between PHA content and total cell productivity was negative (Fig. S4(b)), indicating that the increase in CDW can be attributed to the accumulation of storage compounds. This comes as no surprise, since the PHB content of *C. necator* can reach values up to 90%_CDW_ [28]. Conversely, the PHA and neutral lipid content of *K. phaffii* increased with growth rate (Fig. 3). At the same time, carbohydrate content generally increased with rising growth rate for both organisms, but this correlation’s strength in *C. necator* varied depending on the limiting substrate (Fig. 3(b), bottom). Similar to the correlation between growth rate and protein content, literature reports contradicting relationships between carbohydrate content and growth rate. For instance, in carbon-limited *A. calcoaceticus* the lipid and carbohydrate content remained unchanged at different growth rates [30]. In contrast, du Preez et al. (1984) showed that the carbohydrate content of the carbon-limited *A. calcoaceticus* increased with increasing growth rate. It is important to note that the intracellular storage compound accumulation is interwoven with the nature of the limiting substrate and the capacity of the microbial species to accumulate them. For instance, the carbohydrate content of 5 cultures (*M. extorquens*, *C. glutamicum*, *C. saturnus*, *M. pulcherrima*, and *W. anomalus*) varied between 3.7 and 59%, depending on the microorganism and the limiting substrate (carbon and/or nitrogen) [23].

The growth rate was also correlated with changes in cell size. In general, the cells became larger with increasing growth rate for both organisms, with a significant correlation observed in *K. phaffii* (Fig. 3). Further analysis revealed additional trends when splitting the data into substrate-abundant and substrate-limiting conditions. For example, under substrate-abundant conditions, the cell size of *C. necator* increased with rate (Fig. 3(b), top), while under nitrogen-limiting conditions, the cell size decreased with increasing growth rate (Fig. 3(b), bottom). Overall, nitrogen limitation led to larger cells and higher PHA content and neutral lipid compared to the other tested cases in *C. necator*, and during concurrent limitation of carbon and nitrogen the macromolecular composition of the cells was within the limits established by the limitation of one substrate (Fig. S2).

### The size of a microbial cell is correlated to its macromolecular composition

Microbial cells increase in size during the lag phase, after which they start dividing, and their size subsequently reduces when the growth rate decreases. Here, the size of *C. necator* and *K. phaffii* cells increased at higher growth rates (Fig. 3). This confirms a general observation that during steady-state exponential growth, the mass and volume of a microbial cell increase exponentially with time [31]. Under substrate-limiting conditions, the size of *C. necator* cells presented a positive correlation with growth rate for carbon- and concurrently carbon and nitrogen-limited cells while this correlation was negative for nitrogen-limited cells (Fig. 3(b), bottom). The latter is the result of PHA accumulation which leads to larger cells [32] and, evidently, has a stronger impact on the cell size than the increase that is ascribed to the growth rate increase. A smaller increase in cell size under carbon limitation, compared to nitrogen limitation, was also observed in *C. utilis* [25]. This is an expected effect of nitrogen limitation which is known to trigger the accumulation of intracellular storage compounds, typically resulting in larger cells [32].

The most notable correlation for both organisms was found between cell size and NA content, where larger cells had higher content than smaller ones regardless of the substrate concentration (Fig. 3). The protein content had a weak positive correlation with cell size (Fig. 3(a)), with more notable trends observed when examining each limiting substrate separately. For carbon-limited *K. phaffii* the protein content increased by 10% with a 16% increase in cell size, and for concurrently carbon and nitrogen-limited cells of *C. necator* the protein content increased by 12% with a 7% increase in cell size (Fig. 3(b), bottom). On the other hand, nitrogen limitation alone led to a decrease in protein content for *C. necator* and there was no relationship between protein content and cell size for carbon-limited cells. The negative correlation between the cell size and protein content agrees with the findings of Simon and Azam [33], who noted that when cells become smaller the protein-to-cell ratio increases. In summary, we have hinted that smaller cells have higher nutritional value than larger ones, but more work is needed to verify that claim.

In line with the protein content, carbohydrate, PHA, and neutral lipid content did not present a consistent trend across all substrate limitations (Fig. 3). The carbohydrate content increased (3–31%) with increased cell size (7–16%) for carbon-limited and carbon and nitrogen-limited *C. necator* and *K. phaffii* cells, while larger cells contained less carbohydrates when they were nitrogen-limited (Fig. 3(c), bottom). The PHA and neutral lipid content decreased with increasing cell size for *C. necator* under carbon and concurrent carbon and nitrogen limitation and increased when cells were nitrogen-limited. The PHA and neutral lipid content of carbon-limited *K. phaffii* cells slightly increased with increased cell size.

### Microbial protein quality is related to the growth rate

#### Proteome allocation changes with growth rate

**Fig. 4:**
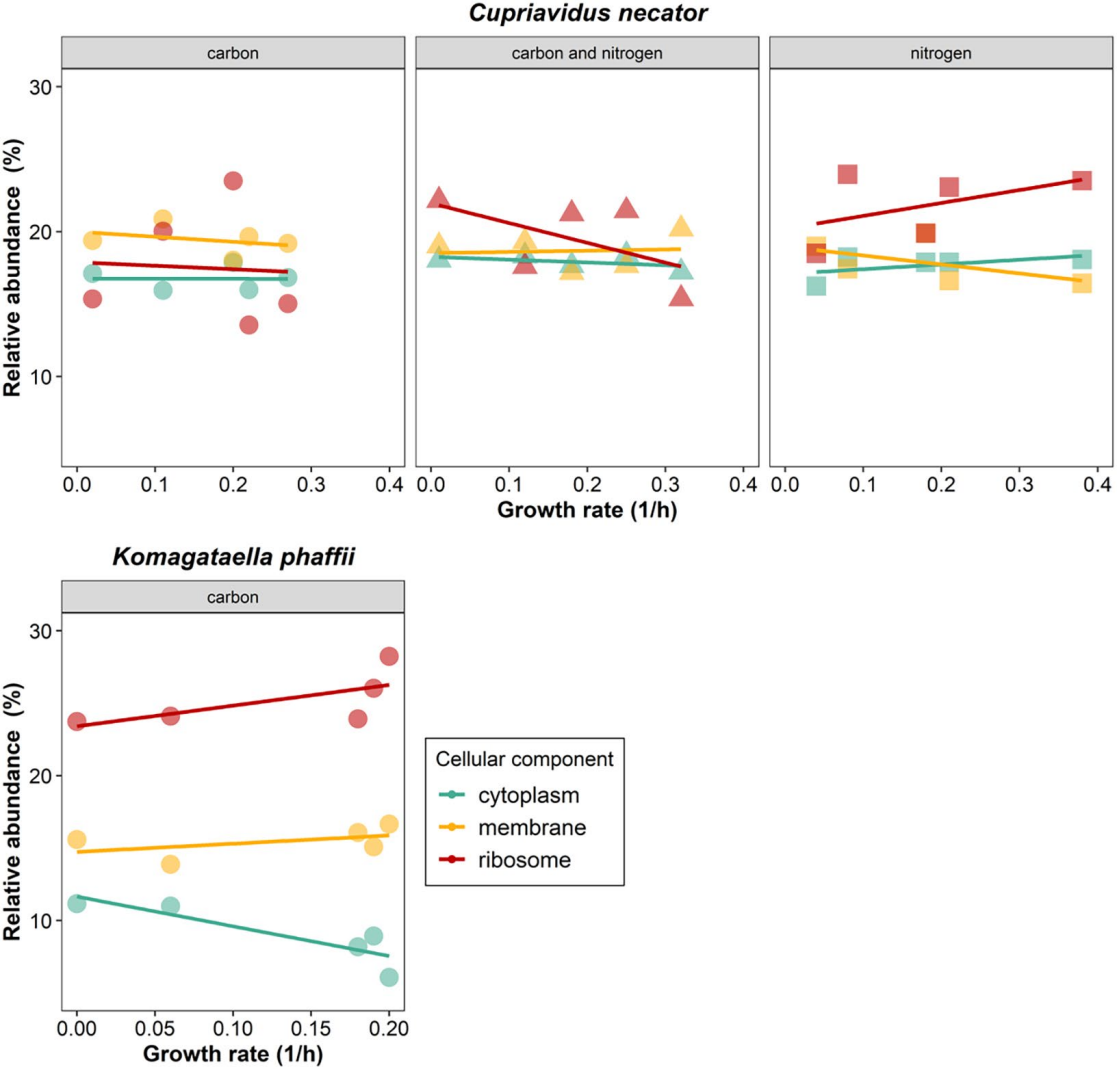
Changes in the proteome allocation across different observed growth rates for *C. necator* (**a**) – (**c**) and *K. phaffii* (**d**). The top 3 cellular components contributing the most to the protein content (based on razor protein intensity) were considered, i.e. cytoplasm, membrane and ribosome

Changes in the environmental conditions trigger adjustments in the metabolic functions of microbial cells (e.g. more abundant substrate leads to higher growth rate at normal cultivation conditions), which in turn cause the necessary proteome reallocation (e.g. more ribosomal proteins at higher rates) [34, 35] so that the microorganisms optimize their fitness. Our analysis showed that ribosomal (14–28%), membrane-related (14–21%) and cytoplasmic (6.1–18%) proteins contribute to up to 60% of the proteome of the two strains considered, and the highest contribution, up to 28%, is ascribed to ribosomal proteins (Fig. 4). In the case of *C. necator*, the contribution of these three cellular components to the protein content was similar (14–24%) and amounted to 49–60% of the total protein, while in the case of *K. phaffii* it was more variable (6.1–28%) and contributed to 48–51% of the total protein (based on razor intensity). Previous work has indicated that from all the cellular components, only ribosomes and chaperones have a considerable protein mass allocated to them in *C. necator* [35]. Here, we observed a general increase in the abundance of ribosomal proteins with increasing growth rate, and a decrease in the cytoplasmic proteins, especially for *K. phaffii* (Fig. 4). Nevertheless, not all proteins contained in the cells could be annotated. Specifically, up to 64% of the protein content is represented by proteins that could be linked to a cellular component (55–64% of the razor intensity for *C. necator* and 53–58% for *K. phaffii*), and the allocation of the remaining unidentified fraction could have an impact on this observation. The ribosomal protein abundance generally increased with increasing growth rate for *K. phaffii*. In the case of *C. necator*, no clear trend could be observed. Specifically, the ribosomal protein abundance increased for nitrogen-limited *C. necator* cells, while it decreased for the carbon- and the concurrently carbon- and nitrogen-limited cells (Fig. 4). The relative abundance of membrane-related proteins did not present a notable variation, while the content of cytoplasmic proteins decreased with increasing growth rate for *K. phaffii* and no notable trend was observed for *C. necator*.

Different microbial proteins have different properties. For instance, RuBisCO proteins (related to carbon fixation) and hydrophobins (fungal proteins) have properties similar to egg albumen [36, 37] and could replace it in meat alternatives [38]. Therefore, changes in the protein profile could affect the intrinsic (i.e. color, taste, texture) and techno-functional (e.g. solubility, thermal activity) properties of the microbial biomass, thereby affecting the quality of the final product and its successful adoption by the food industry as replacement for conventional proteins. Nevertheless, this hypothesis remains to be proven. Information about the content in specific proteins is essential, to determine (or predict) the behavior of microbial biomass or extracted proteins in various food applications (e.g. complement or replace expensive or resource intensive proteins, enable more applications).

#### The amino acid abundance varies across the different cellular components

**Fig. 5:**
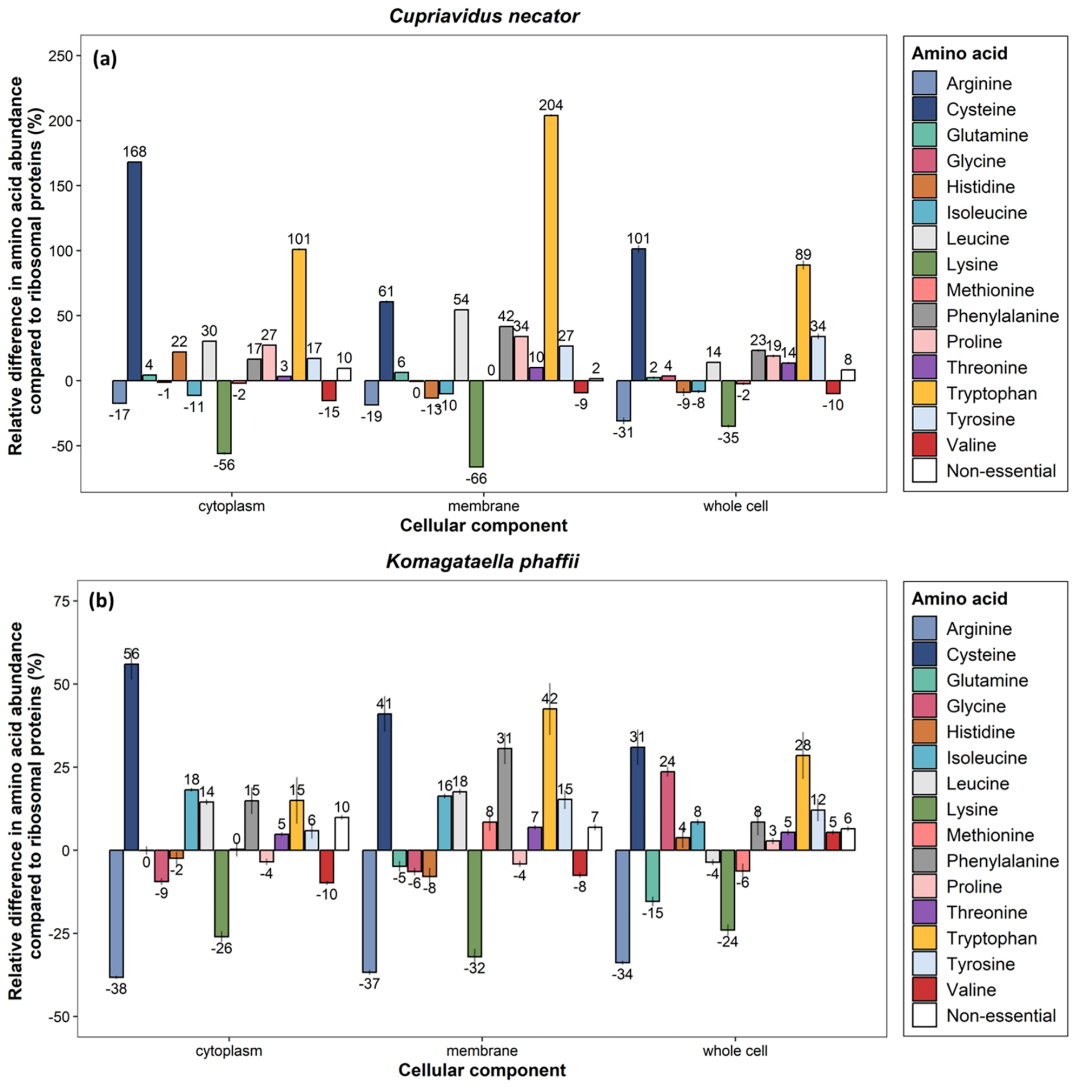
Relative difference in the abundance of essential and conditionally essential amino acids of the 2 cellular components that contribute the most to the protein content and of the whole microbial cells compared to the composition of ribosomal proteins for (**a**) *C. necator* and (**b**) *K. phaffii*. Average values ± standard deviation of all samples are presented

The AA distribution of the whole cells as well as the ribosomal, membrane and cytoplasmic proteins of *C. necator* and *K. phaffii* was established. Next, the relative difference in the AA distribution of the main cellular components and of the whole cells from the AA profile of ribosomal proteins, which contribute the most (14–28%) to the protein content, was calculated (Fig. 5). The AA profile of the different cellular components of *K. phaffii* was considerably less variable compared to *C. necator*, while the differences of the AA profile of the whole cells presented lower deviation from the AA profile of ribosomal proteins compared to the other individual cellular components.

Considering the different AA profile of the proteins contained in the different cellular components [39], the cellular ratio of which is linked to their growth rate [34], it can be expected that the AA profile of their respective cells would be different at different growth rates. Here, we have shown that ribosomal proteins are generally richer in arginine (17–38%), lysine (24–66%), methionine (1.9–6.2%) and valine (7.5–15%) compared to the individual cellular components under investigation as well as the AA profile of the whole cells (Fig. 5). Previous work supports these findings and has shown that ribosomal proteins are richer in these AA compared to metabolic proteins [39]. It can be assumed that the AA profile of ribosomal proteins remains the same since, to cope with the needs of protein production, the cells increase the number of identical ribosomes [40]. Therefore, the number of ribosomes that are linked to the growth rate can substantially impact the abundance of these AA.

Our analysis also showed that cytoplasmic and membrane proteins are generally richer in cysteine (41–168%), leucine (14–54%), phenylalanine (15–42%), threonine (3.3–10%), tryptophan (15–204%) and tyrosine (5.9–27%) compared to ribosomal proteins (Fig. 5). Since cell size (and hence volume) changes depending on the conditions, this may impact the contribution of the cytoplasm, membrane and ribosomes to the final cell volume, and therefore can also impact the cellular abundance of these AA.

#### Amino acid profile changes with growth rate due to different protein allocation

**Fig. 6:**
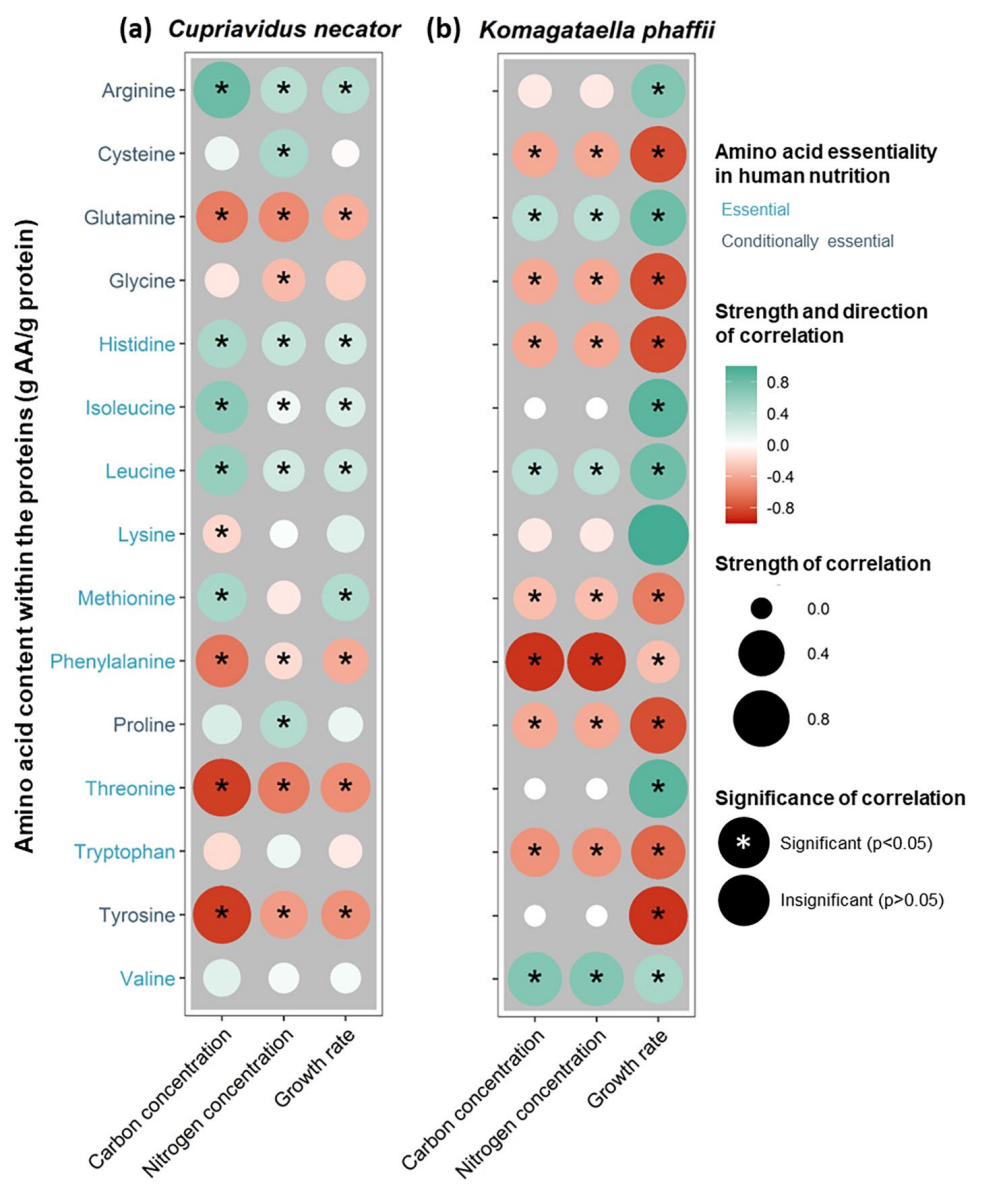
Correlogram for the relationship between content of essential and conditionally essential amino acids for humans (g AA/g protein) in function of carbon (g COD/L) and nitrogen (gNH_4_^+^ /L) concentration and growth rate (h^− 1^) for (**a**) *C. necator* and (**b**) *K. phaffii*

The correlation between substrate concentration, growth rate, and the content of essential and conditionally essential AA for human nutrition in the proteins of *C. necator* and *K. phaffii* was analyzed (Fig. 6). High growth rates yielded microbial proteins rich in arginine, isoleucine, leucine, lysine and valine in both *C. necator* and *K. phaffii*. In contrast, low growth rates resulted in proteins rich in cysteine, glycine, phenylalanine, tryptophan and tyrosine. Therefore, arginine, lysine and valine, which are amongst the AA contained in higher amounts in ribosomal proteins (Fig. 5), were more enriched at higher growth rates. Considering that lysine is commonly limiting in vegetarian diets [41], cultivation at higher rates could result in lower external AA supplementations.

A negative correlation between growth rate and the cysteine content was observed for both *C. necator* and *K. phaffii* (Fig. 6). Methionine content was negatively correlated with growth rate in the case of *K. phaffii* here and in *Enterobacter aerogenes* [40] and *Saccharomyces cerevisiae* [34, 42]. These results hint at the possibility that lower rates result in cysteine- and methionine-rich biomass. Considering that cysteine and methionine are often the limiting AA in microbial biomass [43], cultivation at lower rates could potentially minimize external AA supplementations, nevertheless, more targeted investigation is needed.

Leucine content increased and tyrosine content decreased with increasing growth rate in *C. utilis* proteins [40] while in *S. cerevisiae* contradicting results have been reported for these AA [34, 42]. Similarly, variable relationships between growth rate and the content of glutamine, histidine, methionine, proline and threonine within the proteins of *C. necator* and *K. phaffii* were noted, while *C. necator* was more variable in its response to either carbon or nitrogen availability compared to *K. phaffii* (Fig. 6). These results hint at the employment of different AA biosynthesis pathways for their production and the organism-dependent correlation between growth rate and AA profile. For instance, the negative correlation between the content of eight AA in *S. cerevisiae* was ascribed to the extensive metabolite regulation and the strong feedback inhibition at the AA level during their biosynthesis [34]. Therefore, a general conclusion for the link between growth rate on AA composition across all microorganisms cannot be made. The different effect of rate and substrate concentration (e.g. for lysine in both microorganisms used here; Fig. 6) additionally supports that the limitation of different substrates has a different effect on the AA profile, due to organism-dependent biosynthesis pathways.

## Conclusions

The link between growth rate, substrate availability and the cellular composition and size of *C. necator* and *K. phaffii* was investigated. An increased growth rate was linked to larger microbial cells with an increased NA content and a decreased protein content. Therefore, higher rates result in lower nutritional quality of microbial biomass but could facilitate the biomass harvesting. Furthermore, we showed that the proteome composition is a function of the growth rate, which is also reflected in the AA composition. In summary, we demonstrated tradeoffs between nutritional quality and production rate, which should be considered when designing microbial biomass production processes. Further work should validate and further investigate the links between macromolecular composition and cultivation conditions, as well as their effect on the techno-functional properties of microbial biomass as a food ingredient under industrially-relevant conditions.

### Electronic supplementary material

Below is the link to the electronic supplementary material.

Additional file 1

## Data Availability

Analysis code is available on github.com/CMET-UGent/Sakarika-Kerckhof-et-al-2023. Flow cytometry data were deposited in flowRepository.org (FR-FCM-Z6Y6). The proteomics data have been deposited to the ProteomeXchange Consortium via the PRIDE partner repository with the dataset identifier PXD041491 (username: reviewer_pxd041491@ebi.ac.uk and password: cu2f5CFa). Other data can be provided upon request.
