# Supplementary material for: The nutritional composition and cell size of microbial biomass for food applications are defined by the growth conditions
Source: Microb Cell Fact. 2023 Dec 11;22:254. doi: 10.1186/s12934-023-02265-1 (PMC10712164; doi:10.1186/s12934-023-02265-1)
Supplement: Supplementary file 1 — Additional file 1 [file 12934_2023_2265_MOESM1_ESM.docx]

*Supplementary material for:*

**The nutritional composition and cell size of microbial biomass for food applications are defined by the growth conditions**

Myrsini Sakarika^1,2,†,*^ , Frederiek-Maarten Kerckhof^1,2,3,†^, Lotte Van Peteghem^1,2^, Alexandra Pereira^1,2^, Tim Van Den Bossche^4,5^, Robbin Bouwmeester^4,5^, Ralf Gabriels^4,5^, Delphi Van Haver^4,5,6^, Barbara Ulčar^1,2^, Lennart Martens^4,5^, Francis Impens^4,5,6^, Nico Boon^1,2^, Ramon Ganigué^1,2^, Korneel Rabaey^1,2^

^1^ Center for Microbial Ecology and Technology (CMET), Faculty of Bioscience Engineering Ghent University, Coupure Links 653, 9000 Ghent, Belgium;

^2^ Center for Advanced Process Technology for Urban Resource recovery (CAPTURE), Frieda Saeysstraat 1, 9052 Ghent, Belgium;

^3^ Kytos BV, IIC UGent, Frieda Saeysstraat 1/B, 9052 Ghent, Belgium;

^4^ VIB-UGent Center for Medical Biotechnology, VIB, Ghent, Belgium;

^5^ Department of Biomolecular Medicine, Ghent University, Ghent, Belgium;

^6^ Proteomics Core, VIB, Ghent, Belgium

^*^ Corresponding author: Myrsini Sakarika, Ghent University; Faculty of Bioscience Engineering; Center for Microbial Ecology and Technology (CMET); Coupure Links 653; B-9000 Gent, Belgium; e-mail: [myrsini.sakarika@ugent.be](mailto:myrsini.sakarika@ugent.be).

^†^ Equal contribution

# Supplemental items

# List of figures

To evaluate if bottom-up proteomics yields the same results as a hydrolysate, the samples were analyzed with the gold standard methods by an external accredited commercial laboratory (Euroﬁns Denmark A/S, Denmark). However, this amino acid analysis will not yield results for the biological function of the cultures. Here we have chosen to perform LC-MS bottom-up proteomics as it can provide both the biological function analysis as well as the amino acid analysis.

For the comparison of LC-MS with the results from the external lab, the proteomics data was searched with MSGF+ [1] with the same parameters as mentioned in the main manuscript. The FDR of the search was controlled at 1 % using the target-decoy approach. The search results were then quantified with FlashLFQ [2] with the parameter matching between runs turned on. The quantified proteins were then multiplied with the representation of amino acids in that protein and summed across all proteins for each amino acid separately. The amino acids quantifications were then normalized by dividing by the maximum quantity of the most abundant amino acid. The same normalization was performed for the Eurofins amino acid quantifications.

The comparison between direct amino acid analysis and bottom-up proteomics are shown in Figure S1. The results from the external lab are here considered to be the gold standard. The LC-MS approach shows a high correlation between its quantification results and the gold standard. For the higher abundances the estimated quantity of amino acids with LC-MS is worse than the gold standard, but still sufficient to warrant both the functional and amino acid analysis shown in the manuscript.


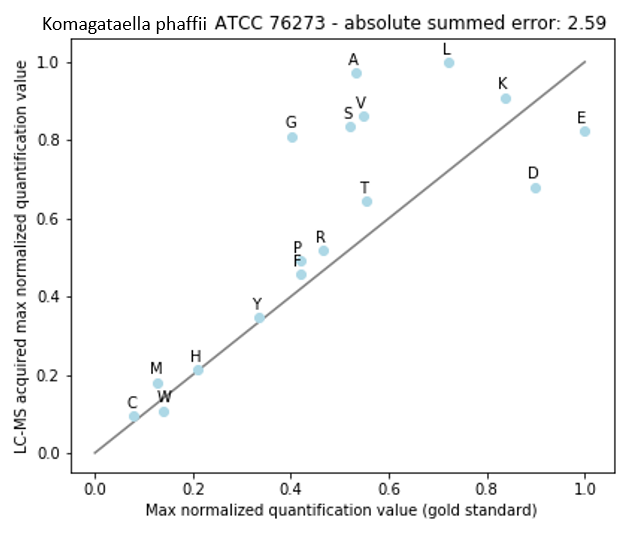

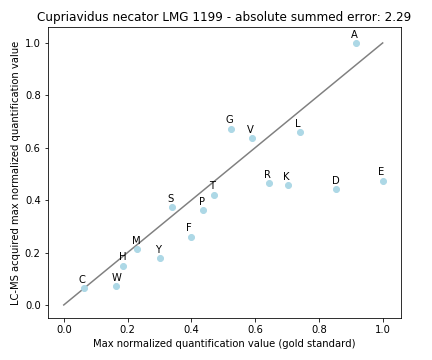


**Figure S1**: Amino acid quantification from a hydrolysate by an external accredited commercial laboratory and a LC-MS approach for bottom-up proteomics. Abbreviations: A = Alanine, R = Arginine, D = Aspartic acid, C = Cysteine + Cystine, E = Glutamic acid, G = Glycine, H = Histidine, I = Isoleucine, L = Leucine, K = Lysine, M = Methionine, F = Phenylalanine, P = Proline, S = Serine, T = Threonine, W = Tryptophan, Y = Tyrosine, V = Valine.


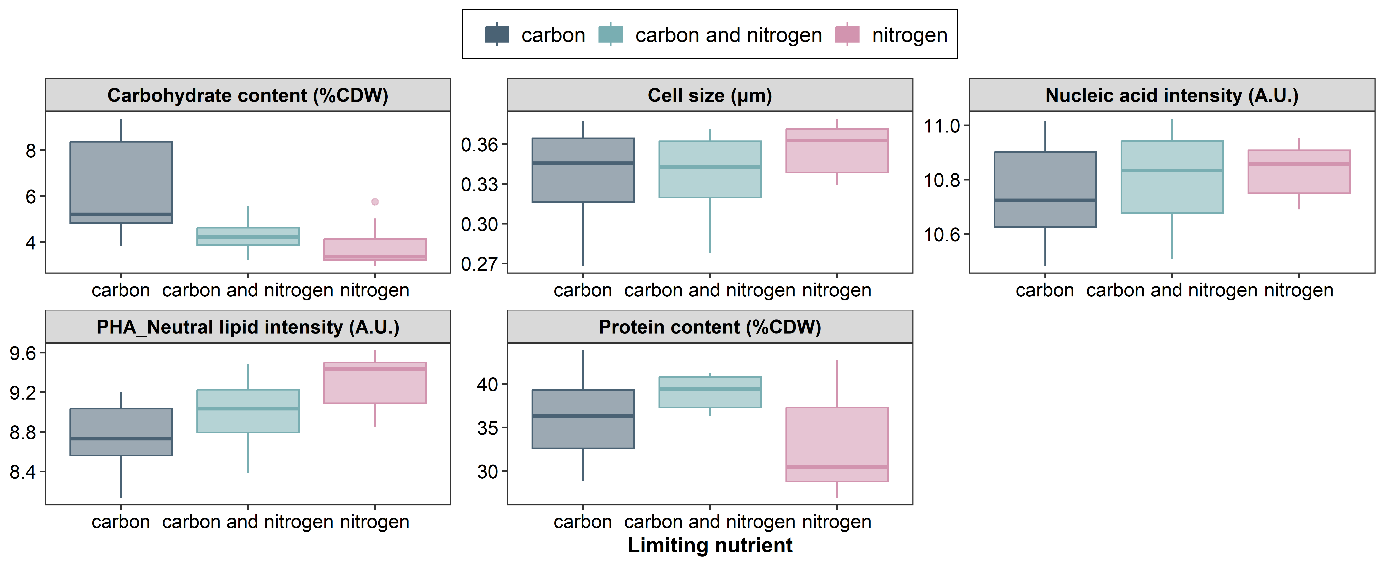


**Figure S2**: Comparison between various parameters and limiting substrate for the substrate-limited cells of *C. necator* (*i.e*. > 14 h of cultivation). Abbreviations: CDW = cell dry weight, A.U. = arbitrary units, PHA = polyhydroxyalkanoate.


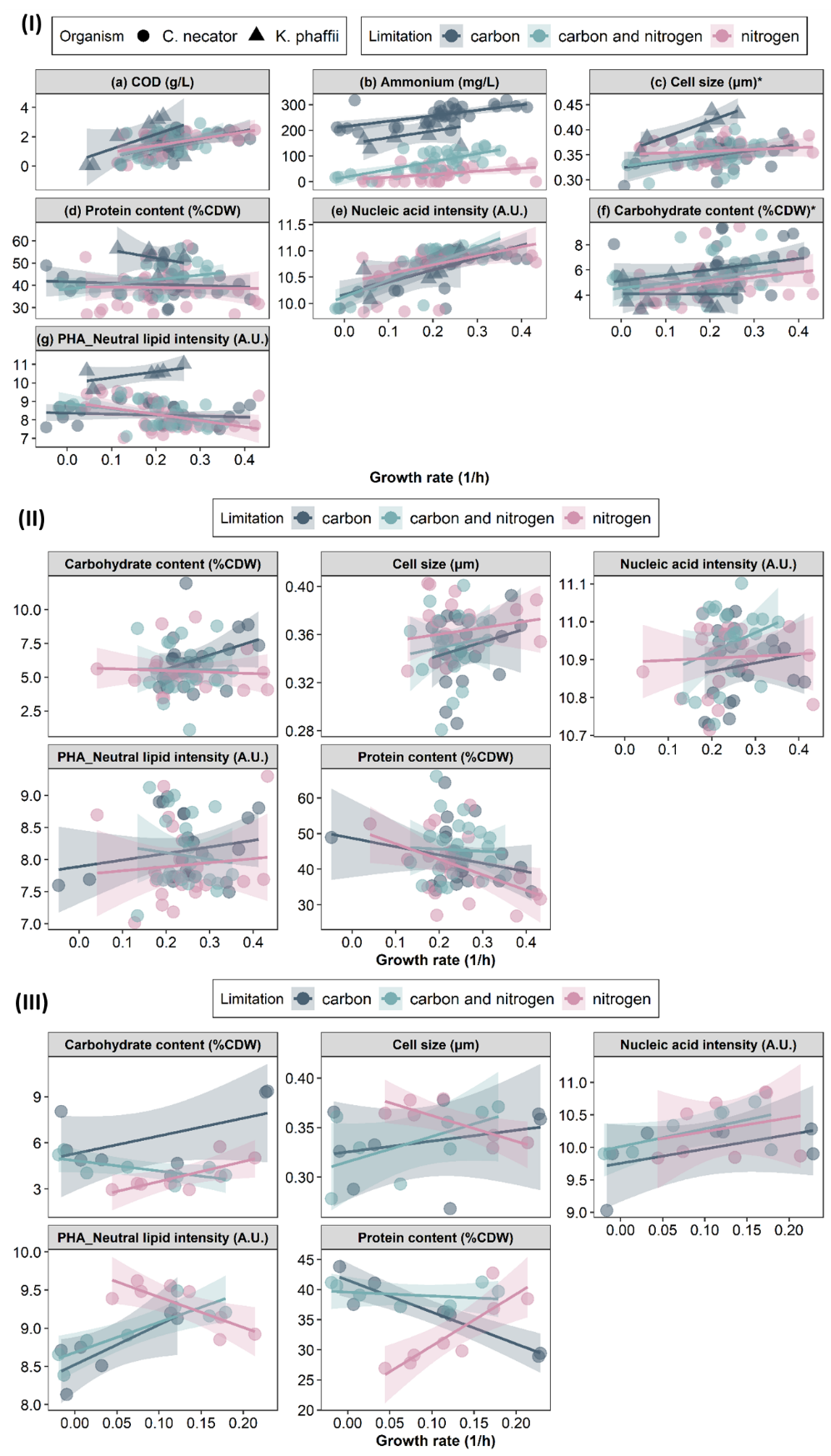


**Figure S3**: (**I**) Macromolecular composition and cell size (diameter) in function of the growth rate throughout the growth of *C. necator* (circles) and *K. phaffii* (triangles). Correlations between growth rate and various parameters for the cells of *C. necator* (**II**) before any limitation (*i.e.* < 14 h of cultivation) and (**III**) for the substrate-limited cells (*i.e.* > 14 h of cultivation). The shaded area indicates the 95% confidence interval of a linear model fit. *The cell size (diameter) and carbohydrate content of *K. phaffii* were divided by 5 to allow for better representation of the trends from both strains in the same graph. Abbreviations: CDW = cell dry weight, A.U. = arbitrary units, PHA = polyhydroxyalkanoate.


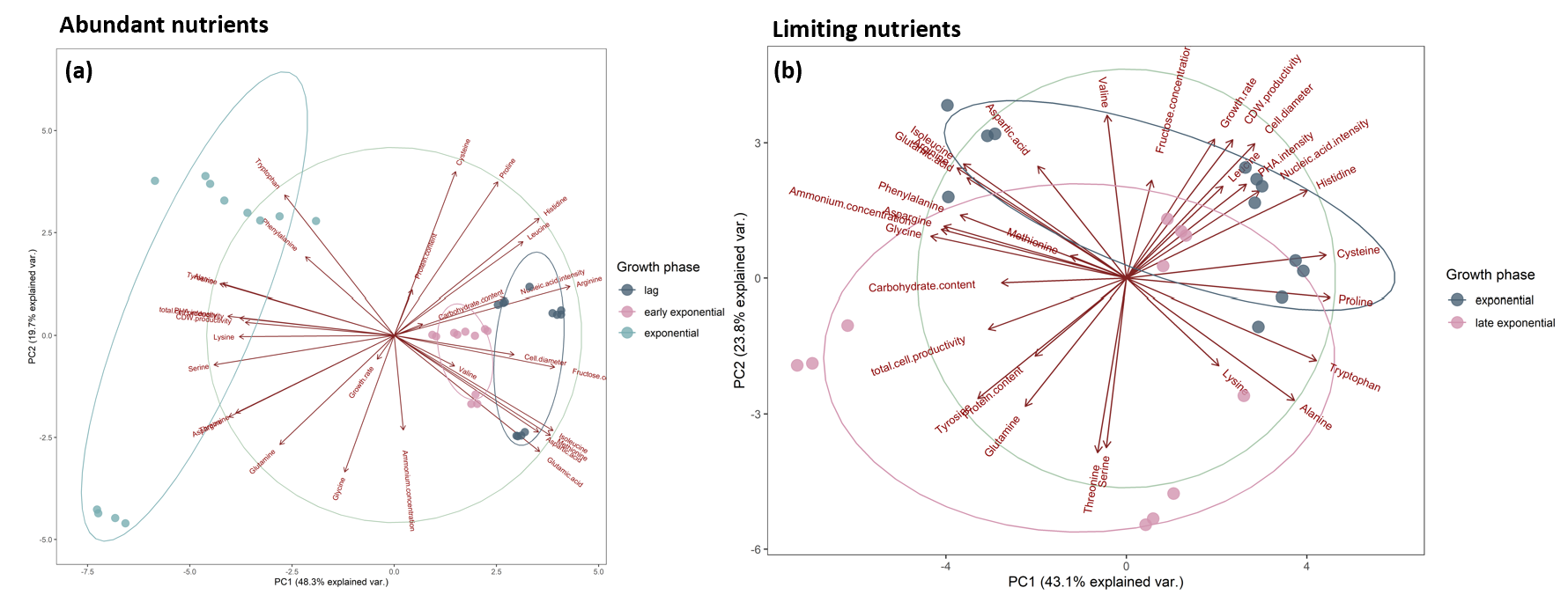


**Figure S4**: PCA-based biplot illustrating the correlations between the different variables (represented as vectors) influencing the microbial protein production process **(a)** when substrates were abundant and **(b)** when substrates were limiting. The length of the vectors represents the contribution of the different variables, and the angle between the vectors indicates how the different variables correlate (*i.e.* small angle = positive correlation; *ca.* 90° = no correlation; large angle = negative correlation). The correlation circle is indicated with green color.


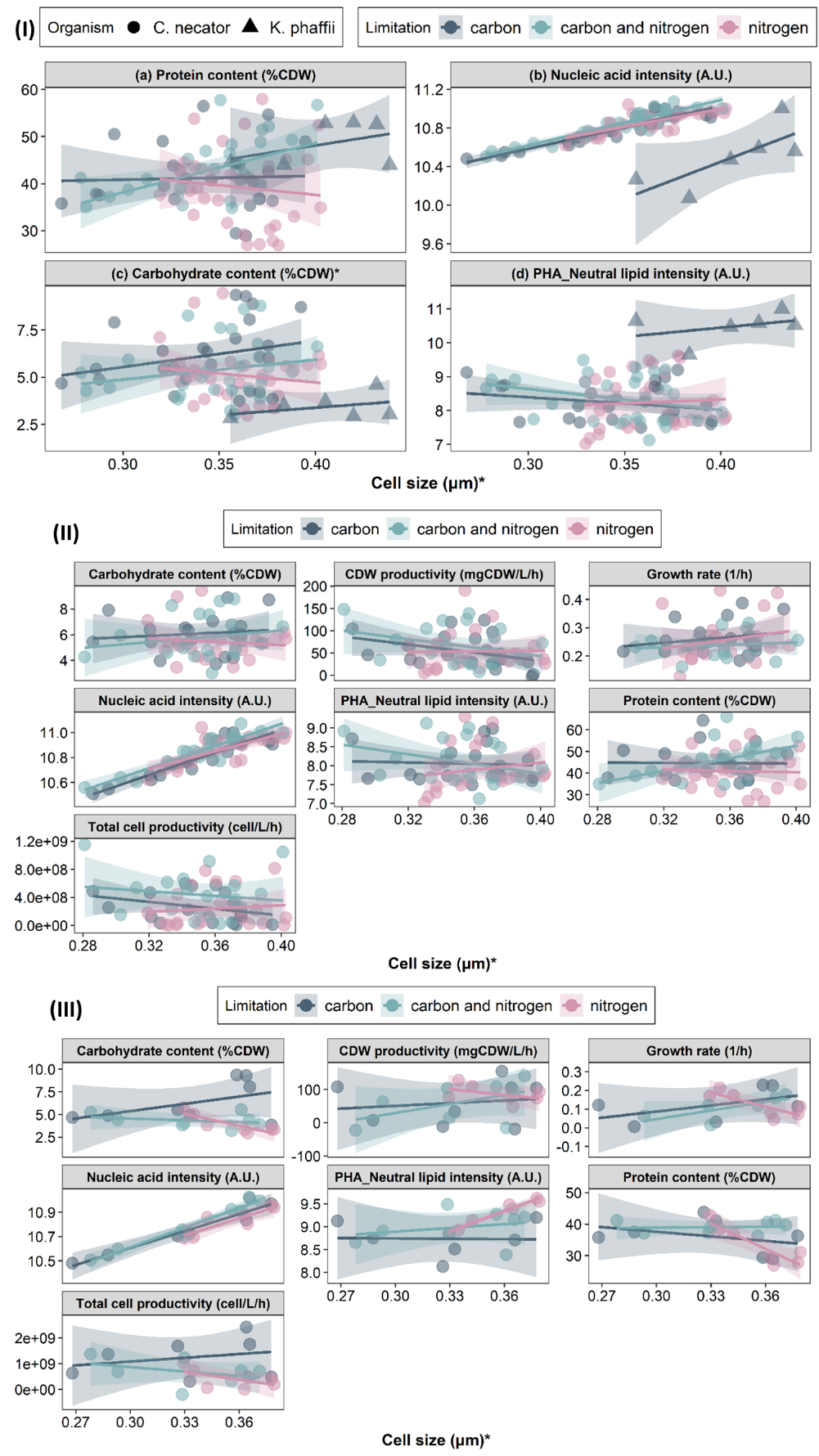


**Figure S5**: (**I**) Macromolecular composition in function of the median cell diameter throughout the growth of *C. necator* (circles) and *K. phaffii* (triangles). Correlations between cell diameter and various parameters for the cells of *C. necator* (**II**) before any limitation (*i.e.* < 14 h of cultivation) and (**III**) for the substrate-limited cells (*i.e*. > 14 h of cultivation). The shaded area indicates the 95% confidence interval of a linear model fit. *The cell diameter and carbohydrate content of *K. phaffii* were divided by 5 to allow for a better representation of the trends. Abbreviations: CDW = cell dry weight, A.U. = arbitrary units, PHA = polyhydroxyalkanoate.

**References**

1. Kim S, Pevzner PA. MS-GF+ makes progress towards a universal database search tool for proteomics. Nat Commun. 2014;5.

2. Millikin RJ, Solntsev SK, Shortreed MR, Smith LM. Ultrafast Peptide Label-Free Quantification with FlashLFQ. J Proteome Res. 2018;17:386–91.
